# Targeting CCL2-CCR2 signaling pathway alleviates macrophage dysfunction in COPD via PI3K-AKT axis

**DOI:** 10.1186/s12964-024-01746-z

**Published:** 2024-07-17

**Authors:** Yue Dong, Ying Dong, Chengyue Zhu, Lan Yang, Hanlin Wang, Junqing Li, Zixuan Zheng, Hanwei Zhao, Wanji Xie, Meiting Chen, Zhijun Jie, Jia Li, Yi Zang, Jindong Shi

**Affiliations:** 1 Department of Respiratory and Critical Care Medicine, Shanghai Fifth People’s Hospital, Fudan University, Shanghai, China; 2 Center of Community-Based Health Research, Fudan University, Shanghai, China; 3 Lingang Laboratory, 100-19 Banxia Road, Pudong New District, Shanghai, 200120 China; 4 State Key Laboratory of Chemical Biology, Shanghai Institute of Materia Medica, Chinese Academy of Sciences, Shanghai, China; 5 School of Chinese Materia Medica, Nanjing University of Chinese Medicine, Nanjing, China; 6 Department of General Medicine, Zhuanqiao Community Healthcare Service Center of Minhang District, Shanghai, China; 7 Department of General Medicine, Hongqiao Community Healthcare Service Center of Minhang District, Shanghai, China; 8 Hangzhou Institute for Advanced Study, University of Chinese Academy of Sciences, Hangzhou, China; 9 Zhongshan Institute for Drug Discovery, Shanghai Institute of Materia Medica, Chinese Academy of Sciences, Guangdong, China; 10 Shandong Laboratory of Yantai Drug Discovery, Bohai Rim Advanced Research Institute for Drug Discovery, Yantai, Shandong China

**Keywords:** Chronic obstructive pulmonary disease, C–C motif chemokine ligand 2, C–C chemokine receptor type 2, Bronchial epithelial cells, Macrophage

## Abstract

**Background:**

Chronic obstructive pulmonary disease (COPD) remains a leading cause of morbidity and mortality worldwide, characterized by persistent respiratory symptoms and airflow limitation. The involvement of C–C motif chemokine ligand 2 (CCL2) in COPD pathogenesis, particularly in macrophage regulation and activation, is poorly understood despite its recognized role in chronic inflammation. Our study aims to elucidate the regulatory role and molecular mechanisms of CCL2 in the pathogenesis of COPD, providing new insights for therapeutic strategies.

**Methods:**

This study focused on the CCL2-CCR2 signaling pathway, exploring its role in COPD pathogenesis using both *Ccl2* knockout (KO) mice and pharmacological inhibitors. To dissect the underlying mechanisms, we employed various in vitro and in vivo methods to analyze the secretion patterns and pathogenic effects of CCL2 and its downstream molecular signaling through the CCL2-CCR2 axis.

**Results:**

Elevated Ccl2 expression was confirmed in the lungs of COPD mice and was associated with enhanced recruitment and activation of macrophages. Deletion of *Ccl2* in knockout mice, as well as treatment with a Ccr2 inhibitor, resulted in protection against CS- and LPS-induced alveolar injury and airway remodeling. Mechanistically, CCL2 was predominantly secreted by bronchial epithelial cells in a process dependent on STAT1 phosphorylation and acted through the CCR2 receptor on macrophages. This interaction activated the PI3K-AKT signaling pathway, which was pivotal for macrophage activation and the secretion of inflammatory cytokines, further influencing the progression of COPD.

**Conclusions:**

The study highlighted the crucial role of CCL2 in mediating inflammatory responses and remodeling in COPD. It enhanced our understanding of COPD's molecular mechanisms, particularly how CCL2's interaction with the CCR2 activates critical signaling pathways. Targeting the CCL2-CCR2 axis emerged as a promising strategy to alleviate COPD pathology.

**Graphical Abstract:**

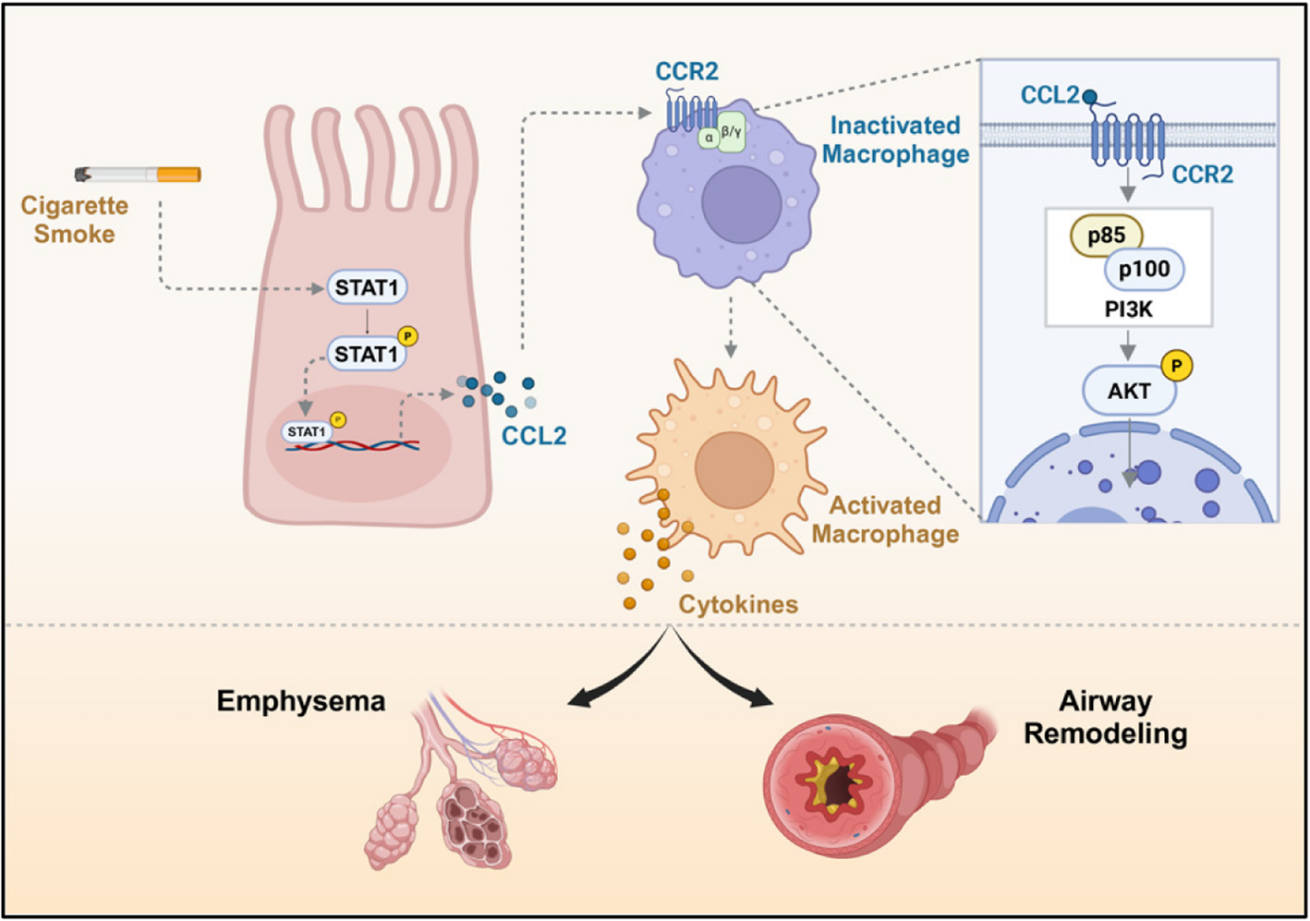

**Supplementary Information:**

The online version contains supplementary material available at 10.1186/s12964-024-01746-z.

## Background

Chronic obstructive pulmonary disease (COPD) is currently the most prevalent chronic respiratory disease, affecting approximately 200 million individuals worldwide [1]. COPD results in approximately 3 million deaths annually, imposing a significant burden on public health [1, 2]. Although current treatments can alleviate symptoms, the combination of inhaled corticosteroids, long-acting muscarinic antagonists, and β-adrenoceptor agonists fails to adequately control exacerbations [2, 3]. The development of novel drugs is urgently needed, which necessitates in-depth research on the pathogenesis of COPD. According to previous reports, chronic inflammatory responses to cigarette smoke (CS) are considered a major contributing factor in COPD development [4–6]. CS contains numerous hazardous chemical compounds, which trigger the pulmonary immune responses and increase cytokines production, especially C–C motif chemokine ligand 2 (CCL2) [7].

CCL2 is the first purified and biologically characterized member of the human CC chemokine family, and it is commonly believed to exert its biological functions through interaction with the C–C motif chemokine receptor 2 (CCR2) [8–10]. Notably, CCL2 levels were significantly increased in sputum from COPD patients compared with healthy volunteers [11]. Elevated levels of CCL2 undoubtedly promote the accumulation of monocyte-derived macrophages locally, a hallmark inflammatory manifestation in the lungs of COPD patients. Yet, CCR2, identified as a G-protein-coupled receptor, has been shown to have a highly diverse downstream pathway, including PI3K/AKT and JAK/STAT pathways, suggesting that the effects of CCL2 on macrophages may extend beyond mere recruitment [12]. Macrophages play a pivotal role in initiating and sustaining this inflammatory status, characterized by their early recruitment and extensive cellular communication [13, 14]. The persistent inflammation, which is dependent on activated macrophages, forms the pathological basis for progressive airflow limitation. During this process, macrophages exhibit a dysregulated cytokine secretion profile, reduced phagocytic capacity for pathogens and apoptotic cells, and a diminished ability to maintain the immune microenvironment [14]. Collectively, these changes are referred to as macrophage functional homeostatic aberrancy, with the altered cytokine profile being particularly indicative. Although several studies have demonstrated that cigarette smoke induces various functional changes in macrophages in COPD patients [15], including alterations in glutamine metabolism [16], phagocytic capacity, and levels of inflammatory cytokine secretion [17], the regulatory role of CCL2 on macrophage homeostasis remains obscure, and its pathogenic contributions to COPD have yet to be comprehensively dissected. Thus, this study aims to clarify the contributions of CCL2 to COPD and dissect the underlying molecular mechanisms, providing theoretical insights for potential clinical applications.

In this study, we confirmed the elevated expression levels of CCL2 in the lungs of COPD by combining bioinformatics and animal experiments. Remarkably, using *Ccl2* deficient mice, we substantiated CCL2's comprehensive contribution to COPD. Delving deeper into the biological processes, we discovered that CCL2 expression originates from bronchial epithelial cells and is regulated by the activation of the STAT1 signaling pathway, which is an upstream mechanism induced by cigarette smoke constituents and lipopolysaccharide. Beyond recruiting macrophages, CCL2 in the pulmonary microenvironment also modulates macrophage homeostasis through the activation of the PI3K-AKT signaling pathway. Ultimately, we conducted prophylactic treatment experiments on mice using compounds that inhibit CCL2-CCR2 signaling, confirming the potential clinical application of our findings.

## Methods

### Cell culture and treatment

The bronchial epithelial cell line BEAS2B was cultured in DMEM (11,965,092, Gibco Life Technologies, Oshima, New York) with 10% fetal bovine serum (26,140,079, Gibco Life Technologies, Oshima, New York) and penicillin–streptomycin (15,140,122, Gibco Life Technologies, Oshima, New York). After growing to 80% confluency, cells were passaged or seeded into 12-well plates, followed by treatment with CSE and LPS for 2 h. Prior to treatment, cells underwent starvation in medium containing 2% FBS for 12 h. If collection of supernatants was required, fresh complete medium would replace the original medium after the 2-h induction period, and conditioned medium was collected after an additional 24 h. Fludarabine (HY-B0069, MCE, Monmouth Junction) was also used to treat BEAS2B cells to inhibit STAT1 phosphorylation, with the treatment duration being the same as that for CSE and LPS.

THP-1 cells were cultured in RPMI media (11,875,093, Gibco Life Technologies, Oshima, New York) + 10% FBS + penicillin–streptomycin and β-mercaptoethanol (M3148, Sigma-Aldrich, St. Louis). Differentiation conditions involved adding PMA (524,400, Sigma-Aldrich, St. Louis) to complete medium for 24h to prime the THP-1 monocytes into macrophage-like cells. We treated THP-1 cells with recombinant CCL2 protein or conditioned medium (CM) for a duration of 24 h. Additionally, cells will be treated with the CCR2 inhibitor INCB3284 (HY-15450A, MCE, Monmouth Junction) and AKT inhibitor perifosine (HY-50909, MCE, Monmouth Junction). The CM would be used to treat the cells at a 1:1 ratio with complete medium. Cells were maintained at 37 °C, 5% CO2 in a humidified culture incubator.

To extract bone marrow-derived macrophages (BMDMs), mice were euthanized, and their femurs and tibiae were collected under sterile conditions. The bones were cleaned of any attached muscles and connective tissues and then flushed with iced PBS using a 27G needle to collect the bone marrow cells. The collected bone marrow cells were passed through a 70 μm cell strainer to obtain a single-cell suspension. Cells were then cultured in IMEM medium supplemented with 10% FBS, penicillin–streptomycin, and 10 ng/mL M-CSF (RP01216, Abclonal, Hubei) for 7 days to differentiate into macrophages. The medium was refreshed every day. After differentiation, BMDMs were treated with recombinant Ccl2 protein.

### Animals

All experimental procedures were approved by the Institutional Animal Care and Use Committee of the Shanghai Institute of Materia Medica (IACUC). Specific-pathogen-free male C57BL/6 mice (20 ~ 25 g, seven to eight weeks-old) and *Ccl2* KO mice were purchased from the Shanghai Model Organisms Center and Jackson Laboratories [18]. They were housed under standard conditions (temperature 22 ± 2℃, humidity 55 ± 5%, 12-h-light/dark cycle) with food and water.

### CS and LPS-induced COPD murine model

The CS was generated from Huangguoshu cigarette, containing 13 mg of tar, and 1.1 mg of nicotine per cigarette. Exposure to CS was conducted using cigarette smoke generator with the default setting. The mice were daily exposed to CS for 1.5 h in the exposure tower for 49 days (NOE-CSM, Tow-Int Tech, Shanghai). LPS (L2637, Sigma-Aldrich, St. Louis) was intratracheal injected (2.5mg/kg dissolved in 50 µL saline) under anesthesia on day 0 and day 14 [19]. The mice were euthanized at day 49. Each mouse underwent pulmonary function testing before euthanasia with Buxco-PFT (PFT, DSI Harvard Bioscience, New Brighton). FinePoint software (DSI Harvard Bioscience, New Brighton) was used to record relevant parameters. In the INCB3284 treatment experiment, all mice were divided into three groups based on body weight, with one subgroup receiving INCB3284 (5 mg/kg per day intraperitoneally), whereas another subgroup received an equal dose of vehicle. INCB3284 was first dissolved in DMSO to prepare a stock solution of 12.5 mg/mL. This stock solution was then diluted with a 0.5% CMC-Na (sodium carboxymethyl cellulose) solution to achieve a final concentration of 1.25 mg/mL.

### Public data collection and processing

We downloaded the raw data of the transcriptome of COPD mouse lung tissues from the GEO database (GSE52509) [20] and converted the SRA files to fastq using the sra-tools. We used the Fastp tool to remove adapter sequences and perform quality control. GRCm39 HISAT2 index was prepared using the reference and annotation files from GENCODE [21, 22]. RNA-seq reads were then aligned with HISAT2 and the SAM files were converted into the BAM and optionally sorted and indexed using SAMtools [23]. Transcript count for each sample was obtained from BAM file with featureCounts [24]. We also downloaded single cell sequencing data (GSE168191 and GSE196638) [25, 26] of the COPD lung tissues and conducted in-depth analysis. All code supporting bioinformatic analysis are available on request.

For the chromatin immunoprecipitation sequencing (ChIP-Seq) analysis, we utilized ChIP-Seq datasets (GSE15353 and GSE31477), which were obtained using STAT1 antibody immunoprecipitation [27, 28]. Raw sequencing reads underwent automatic quality control and trimming using TrimGalore, followed by mapping to the reference genome with Bowtie2 [29]. The resulting BAM files were processed with samtools markdup to remove duplicate reads. Finally, bamCoverage was used to generate BigWig files for signal visualization, applying RPKM normalization [30].

### Differential expression analysis

The raw count data for individual sample was collated into a matrix and normalized with ‘DESeq2’ R package [31]. Differentially expressed genes (DEGs) in COPD tissue and normal tissue were then calculated by algorithm in ‘DESeq2’ package with cutoff values of |log2 fold change|> 0.5 and false discovery rate (FDR) < 0.05.

### Gene Set Enrichment Analysis (GSEA)

To further investigate the biological function of genes, GSEA was used with R package ‘clusterProfiler’ [32]. We obtained significantly enriched Gene Ontology (GO), Kyoto Encyclopedia of Genes and Genomes (KEGG) and Reactome terms and then visualized them in bar plot (|Normalized enrichment score (NES)|> 1, *p*-value < 0.05, and FDR q-value < 0.25). *P* values were adjusted with the BH methods.

### Single cell RNA sequence analysis

Seurat package was used for downstream analysis of scRNA data [33]. Low-quality cells were filtered (expressing fewer than 200 genes, > 15% mitochondrial reads and > 5,000 unique gene counts). Principal component analysis was performed on normalized and scaled data using 4,000 variable genes. The top principal component analyses were used for clustering and visualized using the UMAP or t-SNE algorithm.

### Preparation of Cigarette Smoke Extract (CSE)

Cigarette smoke extract (CSE) was prepared as previously described with minor modifications [34]. The Huangguoshu cigarettes were used for experiments. Cigarette smoke was drawn into a syringe and slowly bubbled into sterile serum-free cell culture media in 15 mL tubes. Four cigarette was used for the preparation of 10 mL of solution. CSE solution was filtered (SLGS033SS, Merck Millipore, Darmstadt) to remove insoluble particles and was designated as 100% CSE solution.

### Enzyme-linked Immunosorbent assay

CCL2 (RK00052, Abclonal, Hubei) and TGF-β (RK00056, Abclonal, Hubei) in conditioned medium were measured using enzyme-linked immunosorbent assay (ELISA) kits according to the instructions.

### Western blot

Cells were lysed in RIPA buffer (P0013B, Beyotime, Shanghai) containing a protease inhibitor cocktail (HY-K0010, MCE, Monmouth Junction) and 1 mM sodium orthovanadate (HY-D0852, MCE, Monmouth Junction). As for animal sample, approximately 10–20 mg of tissue was suspended in 150 ml RIPA buffer with protease inhibitor cocktail and grinded in a homogenizer (KZ-III-FP, Servicebio, Wuhan) following the manufacturer’s instructions. Samples were centrifuged at 12,000 rpm for 10 min at 4 °C. Protein concentrations were determined by the BCA protein assay kit (P0009, Beyotime, Shanghai). Protein lysates (20μg) were resolved by Tris–Glycine SDS-PAGE and transferred to nitrocellulose membranes (Millipore, Billerica). All membranes were incubated with the indicated primary antibodies overnight at 4°C and were diluted in TBST (20 mM Tris pH 7.5, 150 mM NaCl, 0.1% Tween-20) supplemented with 5% bovine serum albumin (A9418, Sigma-Aldrich, St. Louis). Primary antibodies were diluted 1:1000 in 5% BSA/TBST (except GAPDH, 1:10,000), and secondary antibodies were diluted 1:2500 in 5% BSA/TBST. Primary antibodies were detected with horseradish peroxidase-conjugated secondary antibodies followed by exposure to ECL reagents (SQ201, Epizyme, Shanghai), and densities of bands were quantified by Image J software. The catalog of antibodies used is detailed in Table S1.

### H&E, masson staining, and immunostaining

Lung tissue samples were exposed to fixative, dehydrated with alcohol and xylene, embedded in paraffin, and sliced into sections with a thickness of 4 μm using a paraffin microtome. The sections stained with hematoxylin & eosin (H-E) and masson according to conventional protocols for histopathological evaluation. Measurements of alveolar enlargement were estimated using H&E-stained slides. Microtome sections from H&E-stained sections of paraffin embedded mouse lungs were digitally imaged at 200X magnification (Vectra, ParkinElmer, Waltham). Several random fields were evaluated by a blinded investigator using an image analysis software (Image J), and alveolar enlargement was estimated using the mean linear intercept (MLI) method. Under higher magnification (400X), random independent alveolar walls were selected for measurement. The average of these measurements was used as the indicator for assessing alveolar wall thickness. Airway wall fibrosis was assessed by the presence of thick collagen bundles stained by the masson stain. Three random airways per mouse were evaluated by investigators. Immunofluorescence staining was performed using the Novo-Light 4-plex IHC Kit (M-D110041, WiSee Biotech, Shanghai), following the manufacturer's instructions. The catalog of antibodies used is detailed in Table S1.

### RNA purification and polymerase chain reaction

Approximately 20 mg of lung tissue was suspended in TRIzol (15596026, Invitrogen, Waltham) and then broken mechanically with homogenizer. To allow for complete lysis, the tube was placed on ice and left undisturbed for one hour. For cells, they were directly treated with TRIzol. The subsequent purification process was carried out according to the instructions. Once the RNA purification was complete, we assessed the purity and concentration of the RNA using NanoDrop spectrophotometer (ThermoFisher, Waltham). For each sample, 1 μg of total RNA was reverse transcribed into cDNA using ABScript III RT Master Mix for qPCR (RK20428, Abclonal, Wuhan). The amplification step was completed by use of Universal SYBR Green Fast qPCR Mix (RK21203, Abclonal, Wuhan). The 2^−ΔΔCT^ method was used to calculate the results. The primer sequences were shown in the Table S2.

### Flowcytometry

THP-1 cells were detached and pelleted by centrifugation at 300g for 5 min and washed twice with phosphate-buffered saline (PBS) to remove any residual media or dissociation solution. Subsequently, the cell pellet was resuspended in flow cytometry staining buffer (PBS containing 2% fetal bovine serum) to a concentration of approximately 1 × 10^6^ cells/mL. Each cell suspension was aliquoted into flow cytometry tubes, with each aliquot containing approximately 1 × 10^5^ cells. Cells were stained with CD86-APC and CD206-PE antibodies for 30 min at room temperature in the dark. Following staining, cells were washed twice with staining buffer to remove unbound antibodies, resuspended in 400μL of staining buffer, and kept on ice until flow cytometric analysis. Flow cytometric data acquisition was conducted on a flow cytometer and analyzed with FlowJo.

### Transwell assay

In the Transwell migration assay, 1 × 10^5^ cells were seeded into the upper chamber of a Transwell insert (3422, Corning, New York). The lower chamber was filled with a 1:1 mixture of complete medium and CM. After 24 h of incubation to allow for cell migration, cells remaining on the upper surface were gently removed using a cotton swab and cells on the lower surface of the insert were fixed and stained for counting.

### Statistical analysis

Statistical analyses per experiment are indicated in figure legends. Pairwise comparisons were conducted using Student’s two-tailed unpaired t-test. For multiple comparisons, we performed one-way analysis of variance (ANOVA) followed by Dunnett’s correction to control for Type I error. For data that did not meet normality and homogeneity of variance assumptions, we conducted Mann–Whitney U tests for pairwise comparisons and Kruskal–Wallis tests followed by Dunn’s multiple comparison test for multiple group comparisons. Statistical tests were performed in GraphPad Prism 9.0. A *p* value less than 0.05 was considered significant.

## Results

### CCL2 is highly expressed in COPD lungs

**Fig. 1:**
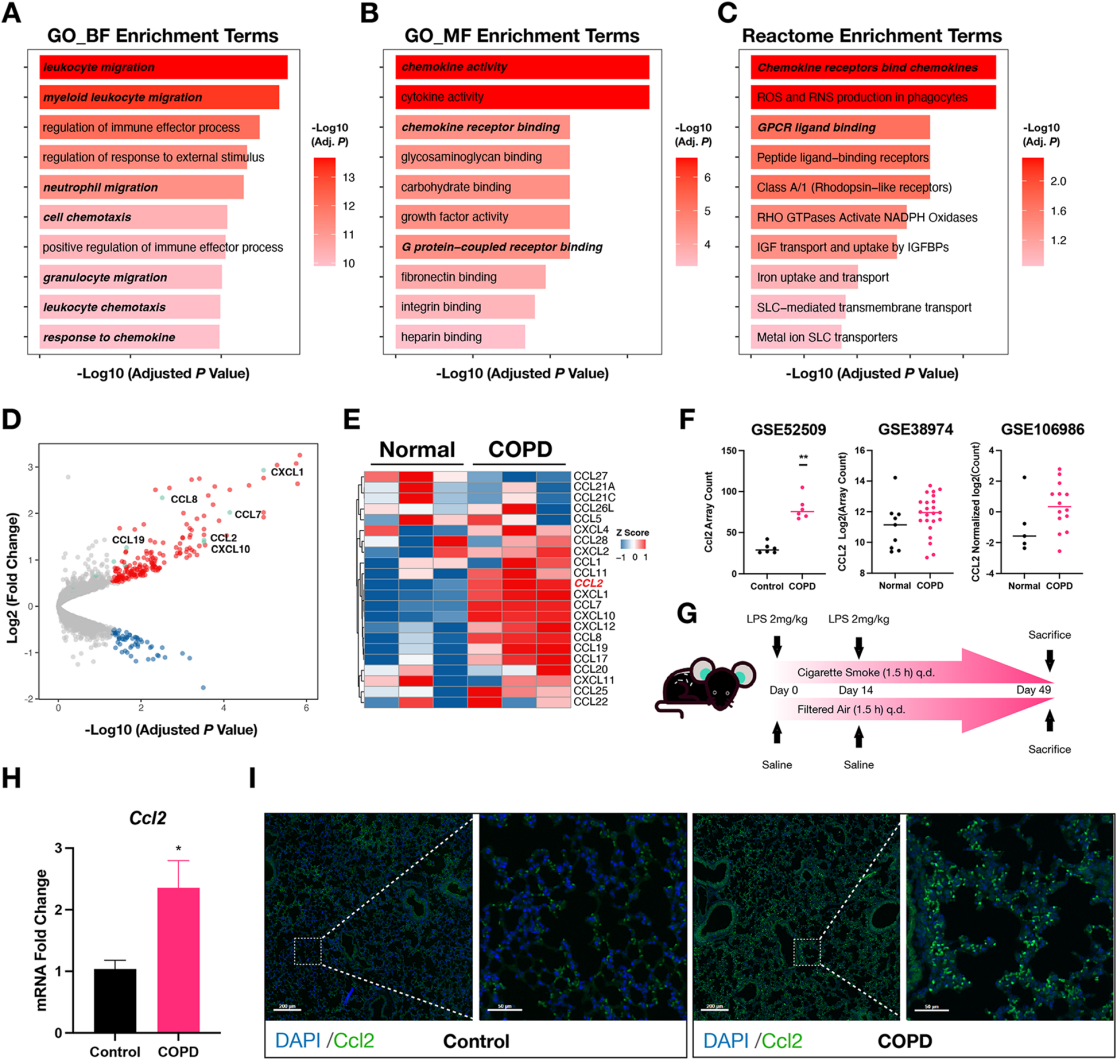
CCL2 is highly expressed in COPD lungs. **A**-**C** Visualization of differential gene enrichment analysis in lung tissues from COPD and normal groups. The gene sets used for enrichment were from GO (**A** and **B**) and Reactome (**C**) databases. Length of bar indicating the magnitude of the adjusted *p* value: longer bars indicate lower adjusted *p* values. The color intensity of the bars deepened with decreasing adjusted *p* values, highlighting more significant enrichment. **D** Volcano plot illustrating DEGs between COPD and normal groups. Red points indicated genes significantly upregulated, blue points represented significantly downregulated genes, and green points were chemokine genes of interest. **E** Heatmap displaying the expression patterns of chemokine profiles between COPD and normal groups. Data have been normalized using the 'scale' command, facilitating comparison across samples. **F** Scatter plot illustrating the differential mRNA levels of *CCL2* between COPD and Normal groups across three datasets: GSE52509, GSE38974, and GSE106986. Horizontal lines represented the mean values. Significance was determined through t-tests, with notations indicating levels of significance: * for *p* < 0.05, ** for *p* < 0.01, *** for *p* < 0.001, and **** for *p* < 0.0001. The absence of a marker denoted a lack of significance. This notation method was consistent for all depicted data. **G** Schematic diagram of the experimental method of COPD modeling. **H** Column plot comparing *Ccl2* mRNA levels in lung tissues between COPD model mice and control mice. The height of each column represented the mean mRNA level. Error bars indicated the Standard Error of the Mean (SEM), highlighting variability within each group. FA: Fresh air; CS: Cigarette smoke. **I** Immunofluorescence assay images comparing Ccl2 fluorescence intensity between two groups. Green signals indicated CCL2, while blue signals represented DAPI, marking the nuclei. Scale bar in low magnification view represented 200 μm, and in high magnification views, the bar represented 50 μm

To identify the characteristically aberrant molecular signaling during COPD pathogenesis, we first conducted an analysis of RNA-Seq data which obtained from the GSE52509 dataset. The DEGs were enriched in various chemokine-related pathways from both the GO and Reactome databases (Fig. 1A-C). Analysis of chemokine profile revealed a notable upregulation of *Ccl2* in COPD lung tissues (Fig. 1D-E). Protein–protein interaction (PPI) network analysis, which was conducted using the STRING tool, revealed that *Ccl2* occupies a central position in the network. This underscores the potential pivotal role of CCL2 in the characteristic molecular network of COPD, highlighting its importance in the biological processes (Figure S1A). Overexpression of *CCL2* was also observed in other human-derived COPD datasets (Fig. 1F). Interestingly, in addition to *Ccl2*, the expression of *Ccl7*, *Cxcl1*, *Cxcl10*, and *Cxcl12* also showed significant increases and occupied central positions in the PPI network of the GSE52509 dataset. However, subsequent analysis of the additional validation datasets revealed that the expression levels of *CCL7*, *CXCL1*, and *CXCL10* did not show changes as significant as those observed for *CCL2* (Figure S1B-C). Conversely, *CXCL12* exhibited a notable downregulation in the COPD group (Figure S1B-C). These findings further solidify our belief in the potential functional importance of *CCL2*.

Following this, we experimentally confirmed the upregulation of *Ccl2* in the lungs of mice with COPD induced by CS exposure and LPS injection. Each mouse underwent daily CS exposure for 49 days and intratracheal injection of LPS on days 0 and 14 (Fig. 1G). The overexpression of *Ccl2* was noted in the COPD mice (Fig. 1H). Together, the immunofluorescent staining photographs showed upregulation of Ccl2 protein levels in the lungs originated from COPD mice (Fig. 1I). These results provided compelling evidence for the increased expression of CCL2 in COPD lung tissues.

### Ccl2 deficiency protects mice against CS- and LPS-induced alveolar injury and airway remodeling

**Fig. 2:**
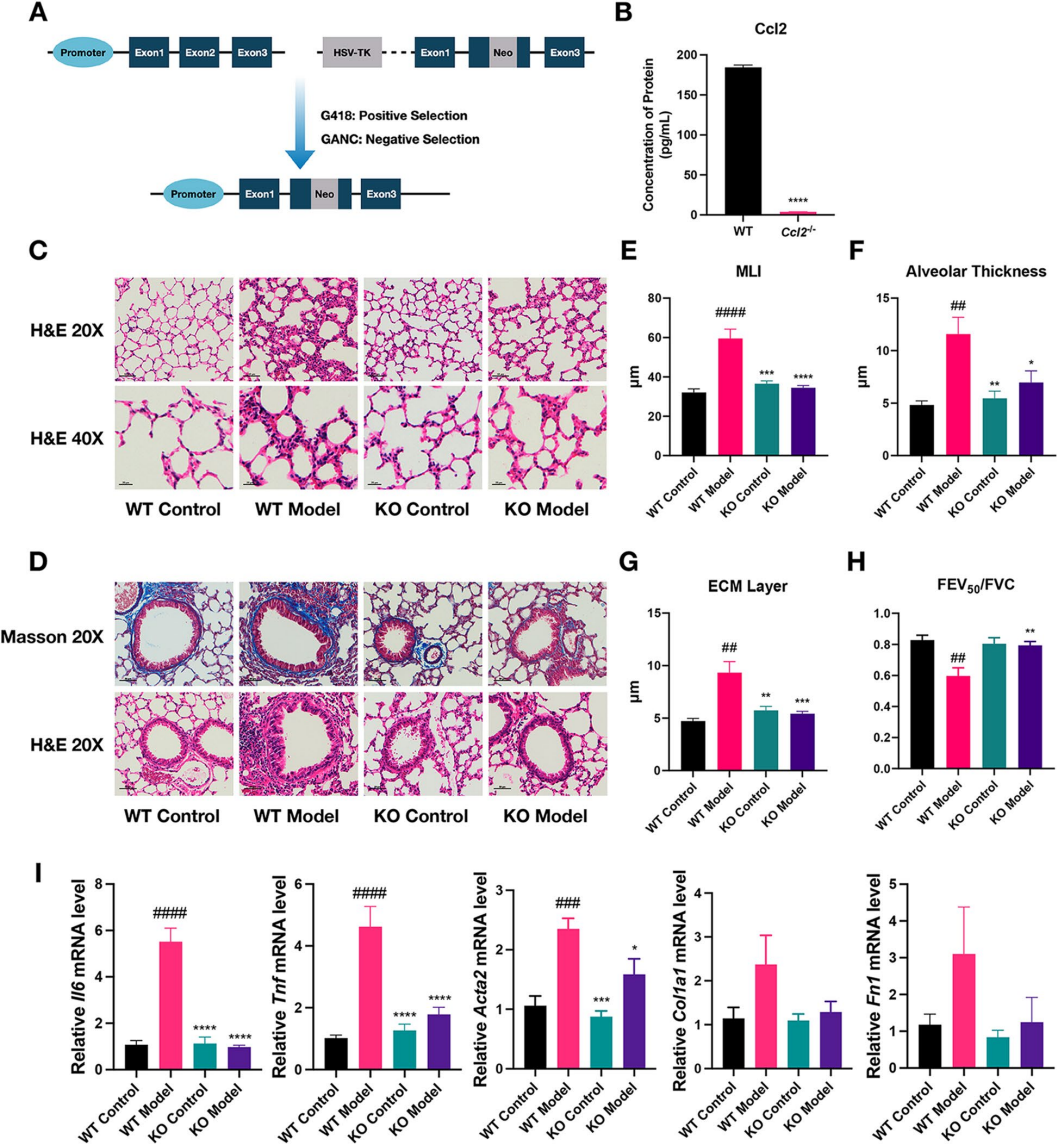
Ccl2 deficiency protects mice against CS- and LPS-induced alveolar injury and airway remodeling. **A** Schematic diagram of *Ccl2* knockout mice construction. **B** Column chart comparing Ccl2 protein levels in lung tissues between wild-type (WT) mice and *Ccl2* knockout (KO) mice, as measured by ELISA experiments. Error bars indicated SEM within each group. **C** Photos of H&E-stained lung tissue sections from WT and KO mice, both with and without COPD model induction. The images were presented at two magnifications: 20X, where the scale bar represented 50 μm, and 40X, where the scale bar represented 20 μm. **D** Photos of H&E- and Masson-stained lung tissue sections from WT and KO mice, both with and without COPD model induction. The scale bar represented 50 μm. **E**, **F** Column plot displaying the MLI (**D**) and alveolar thickness (**E**) levels across four groups. MLI indicated the average size of alveoli. The symbol '#' denoted statistical comparisons between WT Model and WT Control groups, while '*' marked comparisons of other groups against the WT Model group. The number of symbols correlated with the significance of the *p* values, consistent with previous descriptions. **G** Column chart displaying the thickness of ECM across four groups. **H** Column chart depicting the values of FEV_50_/FVC, a ratio of the forced expiratory volume in 50 ms to the forced vital capacity. This ratio served as an indicator of the degree of expiratory limitation. **I** Column graph presented the relative mRNA expression levels of the *Il6*, *Tnf*, *Acta2*, *Col1a1*, and *Fn1* across different groups of mice. Error bars indicated the SEM within each group

To dissect the contribution of Ccl2 to the pathogenesis of COPD, we employed COPD models using *Ccl2* KO mice, while wild-type (WT) mice served as controls. The *Ccl2* KO strain was generated using HSV-TK and neomycin double selection method as previously described [18] (Fig. 2A). *Ccl2* depletion was confirmed by genotyping for the presence of the mutant *Ccl2* allele and WT allele. The *Ccl2* KO and WT mice were subjected to aforementioned COPD modeling procedures. ELISA assays confirmed that Ccl2 was remarkably deleted in the lungs originated from KO mice (Fig. 2B). Moreover, a significant attenuation in alveolar injury and fibrosis along the bronchioles were observed in *Ccl2* KO mice (Fig. 2C-D) as quantified by the lower MLI scores, alveolar thickness and ECM layer thickness (Fig. 2E-G). These histological findings demonstrated the critical role of Ccl2 in COPD pathogenesis. Furthermore, pulmonary function tests demonstrated that airway resistance during the exhalation phase, a key indicator of COPD, was altered. As depicted in Fig. 2H, the FEV_50_/FVC ratio was significantly lower in *Ccl2* KO mice compared to the WT mice, which is the most important functional parameter for assessing the severity of COPD in mice. Consistent with these results, RT-qPCR assay confirmed a significant decrease in the expression *of Il6, Tnf, Fn1, Col1a1* and *Acta2* (Fig. 2I). These markers reflect the severity of pulmonary inflammation and fibrosis. Taken together, our data support that the deficiency of *Ccl2* protects mice against CS/LPS-induced lung injury and remodeling.

### Cigarette components stimulate bronchial epithelial cells to express CCL2 via STAT1 phosphorylation

**Fig. 3:**
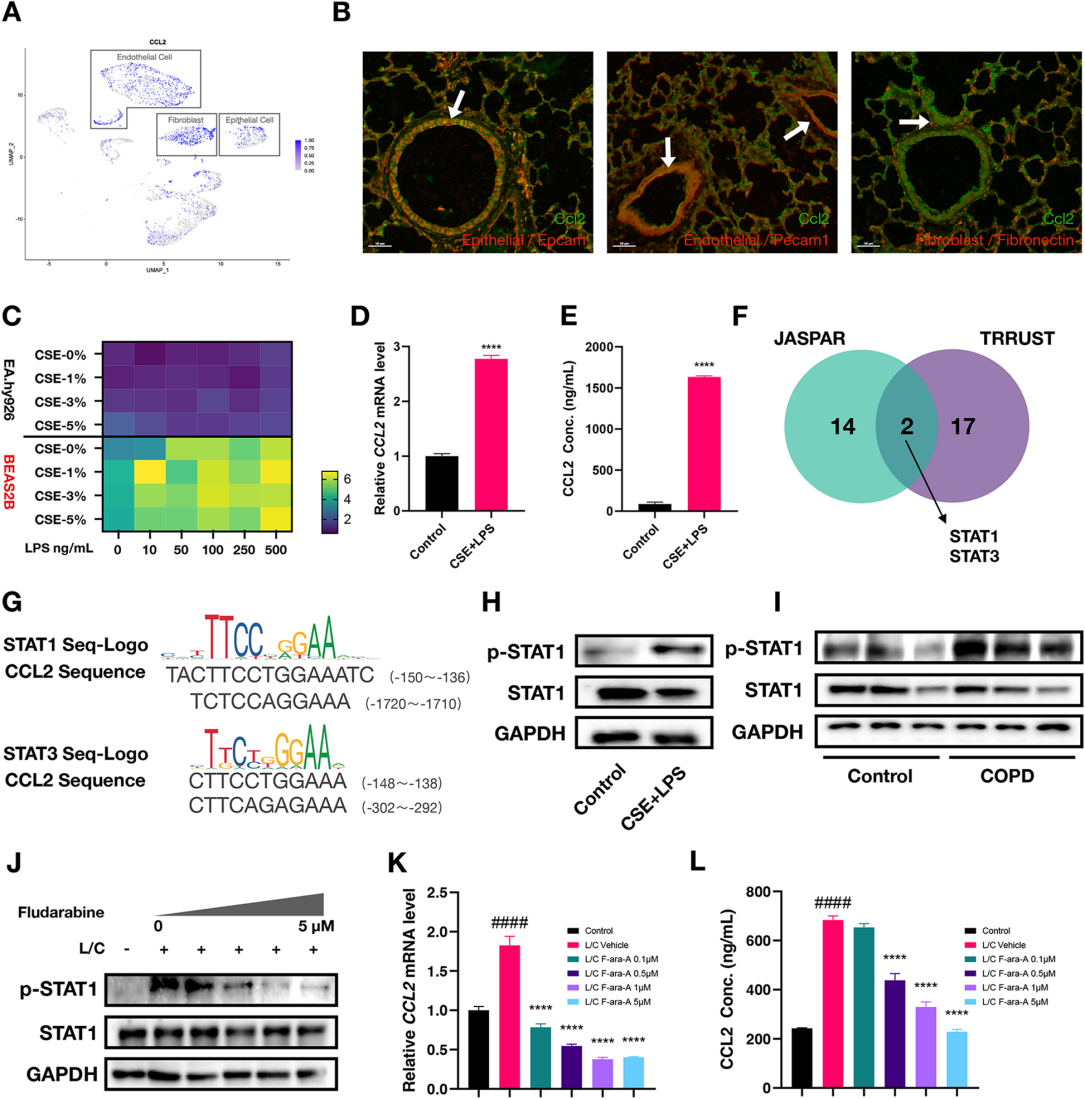
STAT1 contributes to the increased expression of CCL2 in bronchial epithelial cells. **A** Dimensionality reduction plot (Dimplot) showing the spatial distribution of *CCL2* expression from scRNA-Seq data (GSE168191). The intensity of the color within each cell-point correlated with the level of CCL2 expression. Cell cluster annotations were determined based on the results from Figure S2A and S2B. **B** Immunofluorescence staining demonstrating colocalization of Ccl2 with various cell-specific markers. White arrows pointed to cells where colocalization of Ccl2 and cell markers occurs. Scale bar: 50 μm. **C** Heatmap displaying *CCL2* mRNA Levels in two cell types under various concentrations of CSE and LPS Co-Treatment. The reference value for comparison was the untreated EA.hy.926 cells, with color intensity reflecting the fold change in mRNA levels relative to this control. **D**, **E** Column graph comparing the changes in CCL2 mRNA (**D**) and protein levels (**E**) between groups treated with CSE (5%) and LPS (500 ng/mL) versus the control group. **F** Venn diagram showing the transcription factors predicted by JASPAR and TRRUST databases, with STAT1 and STAT3 highlighted as overlapping transcription factors. **G** The alignment between the recognition motifs of STAT1 and STAT3 and the upstream sequence of the *CCL2* transcription start site. (H) Western blot analysis visually quantifies the level of STAT1 phosphorylation in response to treatment with CSE and LPS compared to untreated controls. **I** Western blot experiment comparing changes in phosphorylated STAT1 levels in lung tissues between COPD model mice and control group mice. **J** Western blot analysis investigating the effects of fludarabine treatment on STAT1 phosphorylation levels at different concentrations. L/C represented LPS and CSE treatment. **K**, **L** Column plot showing the impact of various concentrations of fludarabine on CCL2 mRNA (**K**) and protein levels (**L**). L/C represented the group treated with LPS and CSE, while F-ara-A standed for the fludarabine treatments. The symbol '#' indicated comparisons between the L/C group and the control group, and '*' marked comparisons of other groups against the L/C group

Macrophage infiltration is a cytological feature of chronic inflammation in COPD patients [6, 35]. Therefore, the high expression of CCL2, known as a prominent chemokine for monocyte, is likely to mark the initiating phase of this process. However, the characteristics and mechanism of CCL2 expression in the lungs remain to be elucidated. Analyzing the scRNA-Seq data of lung tissues facilitated the identification of *Ccl2* spatial expression pattern, with specific attention given to three cell types: endothelial cells, fibroblasts, and epithelial cells (Fig. 3A, Figure S2A, B). To locate the Ccl2 expression in situ, we performed immunofluorescence staining for selected markers on lung sections. These experiments suggested that Ccl2 was predominantly colocalized with epithelial (Epcam-positive) and endothelial (Pecam1-positive) cells (Fig. 3B). Then, we checked CCL2 expression in vitro by using the BEAS2B and EA.hy-926 cell line. Both cells were stimulated by the combination of LPS and CSE, the supernatant fluid and RNA were collected for the following measurement. The mRNA levels of *CCL2* in BEAS2B cells were significantly higher compared to EA.hy.926 cells (Fig. 3C), thus suggesting that CCL2 was predominantly secreted from bronchial epithelial cells. Furthermore, by comparing the stimulatory effects of various concentrations of LPS and CSE (C/E), we confirmed that treatment with 5% CSE and 500 ng/mL LPS resulted in the most significant induction of CCL2 expression, both at the mRNA and secreted protein levels (Fig. 3D-E, Figure S2C). Therefore, these stimulation conditions were used for subsequent experiments involving BEAS2B cells. These experiments reinforced our hypothesis that the primary damage from cigarette components targets bronchial epithelial cells, and the CCL2 protein they secrete is crucial for the progression of inflammation in the disease. Interestingly, by analysing the features of the heatmap, we observed that LPS significantly affected transcription, while CSE has a notable impact on protein levels (Fig. 3C, Figure S2C). This observation led us to speculate that CSE may influence CCL2 secretion through the enhanced cellular vesicle transport function. Recent studies have also identified and investigated the relationship between CSE and vesicles. Benedikter et al*.* found that CSE can induce a 2.3-fold increase in the release of extracellular vesicles (EVs) from airway epithelial cells [36]. Furthermore, both the lipid profile of the EV membrane and the protein profile inside EV undergo significant changes with CSE exposure [37, 38]. These alterations comprehensively affect the stability, uptake, and biological effects of EVs and may also include an enhancement in the secretion of CCL2, which is crucial for the pathogenesis of COPD.

Transcription factors, as the most direct upstream regulators, are key elements of the molecular pathway through which cigarette components induce the expression of CCL2. We predicted the potential TFs using the JASPAR and TRRUST databases [39, 40]. The overlapping result identified STAT1 and STAT3 as potential upstream factors (Fig. 3F, Table S3). The DNA-binding motif of both factors showed a high affinity for the promoter region of CCL2 (Fig. 3G). Experimental verification revealed enhanced levels of phosphorylated STAT1 (p-STAT1), rather than p-STAT3, in BEAS-2B cells treated with C/E (see Fig. 3H and Figure S2D, E) as well as in the lungs of mice with COPD (Fig. 3I). Additionally, our analysis of ChIP-Seq data revealed a significant peak signal of STAT1 in the promoter region of CCL2 following IFNγ induction, demonstrating the direct interaction between STAT1 and the CCL2 promoter region (Figure S2F). Time-course experiment demonstrated the early characteristic of STAT1 phosphorylation in BEAS-2B cells stimulated with C/L, showing that short-term stimulation induced a rapid increase in CCL2 expression and secretion, but prolonged stimulation reduced its expression and secretion (Figure S2G-I). This activation and secretion timing also align with our hypothesis that bronchial epithelial cells are involved in the early induction rather than the maintenance of inflammation. Fludarabine is recognized as an effective inhibitor of STAT1 phosphorylation. In our experiment, cells were co-treated with C/E and fludarabine simultaneously to investigate the dependency of CCL2 expression on STAT1 activity. The western blot analysis showed a dose-dependent reduction of p-STAT1 in BEAS2B cells (Fig. 3J). In line with the result, RT-qPCR and ELISA assay also confirmed the dose-dependent reduction of mRNA and protein levels of CCL2 in fludarabine-treated BEAS2B cells (Fig. 3K, L). Therefore, these data demonstrated that CS components and LPS primarily induced overexpression of CCL2 specifically in bronchial epithelial cells, which is regulated by phosphorylated STAT1.

### CCL2 induces macrophage migration and activation

**Fig. 4:**
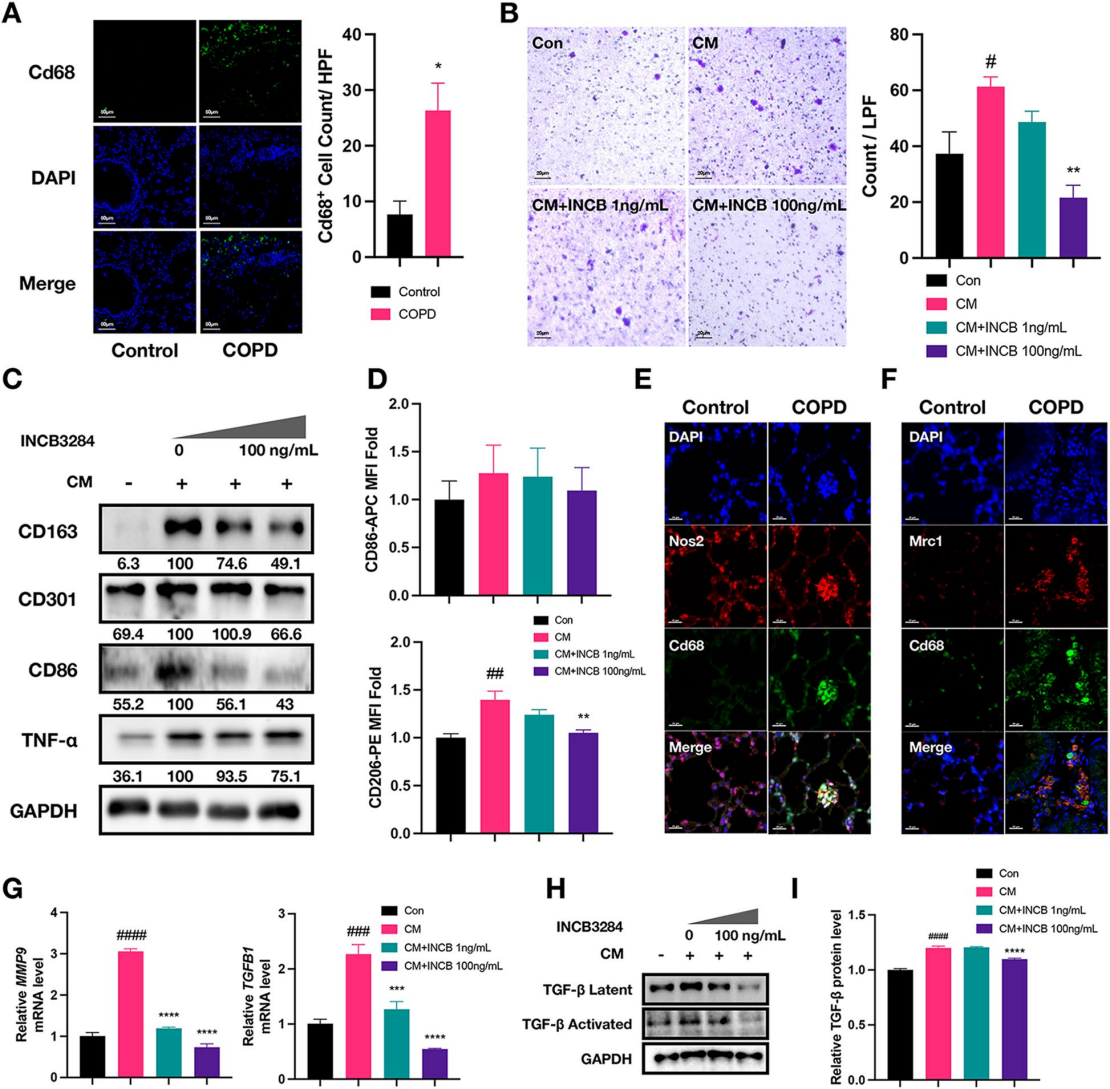
CCL2 induces macrophage migration and activation. **A** Immunofluorescence staining comparison of macrophage Infiltration (Cd68-Positive) in lung tissues between COPD group mice and control group mice. This image analysis quantitatively compared macrophage infiltration levels, using high-magnification views to count Cd68-positive cells in lung tissues of both COPD model mice and their control counterparts. Scale bar: 50 μm. **B** Transwell migration assay images displaying changes in THP-1 cell migration ability after treatment with CM and INCB3284. This image analysis quantitatively compared THP-1 infiltration levels, using low-magnification views to count crystal violet staining-positive cells among the four groups. The symbol '#' indicated comparisons between the CM group and the control group, and '*' marked comparisons of other groups against the CM group. Scale bar: 20 μm. **C** Western blot analysis of activation marker protein levels in THP-1 cells treated with CM and INCB3284. Protein band intensities were normalized to GAPDH as a loading control to ensure accurate quantification. The relative expression levels of each activation marker were calculated as ratios compared to the CM-treated group, utilizing ImageJ for band intensity analysis. **D** Flow cytometry analysis was used to compare changes in the expression levels of CD86 and CD206 across four cell groups. MFI was calculated by FlowJo software. **E**, **F** Immunofluorescence assay analyzed the infiltration of Cd68- and Nos2-positive cells (M1, E) and Mrc1- and Cd68-positive cells (M2, F) in the lungs of control and COPD mice. Scale bar: 50 μm. **G** Column plot showing the *MMP9* and *TGFB1* mRNA levels across four cell groups. **H** Western blot analysis showing the protein levels of latent and activated TGF-β across four groups. **I** ELISA assay showing the secretion levels of TGF-β for each group, with data normalized to the control group to calculate relative levels

Macrophages participate in the pathogenesis of COPD, but the regulatory role of CCL2 on macrophage function has not been well demonstrated. The significantly increased count of Cd68-positive cells in the lungs of COPD mice demonstrated the fidelity of this mouse model in replicating human pathological features (Fig. 4A). Consistently, when conditioned medium (CM) from C/E-treated BEAS2B cells was added to the lower chamber of a transwell insert, it increased the migration of differentiated THP-1 cells (Fig. 4B). Additionally, treatment with INCB3284, a common CCR2 inhibitor, reduced this migratory response, indicating diminished chemotactic activity (Fig. 4B). To eliminate the potential impact of compound cytotoxicity on this experiment, a CCK-8 assay was conducted to assess the effect of INCB3284 on cell viability. The treatment concentration of 100 ng/mL was found not to exhibit significant cytotoxicity (Figure S3A), demonstrating that INCB3284 inhibited CM-induced cell chemotactic activity rather than causing an artifact of inhibition due to potential cytotoxicity. In summary, the results confirmed the effect of CCL2 on macrophages recruitment.

Activated by specific stimuli, macrophages exhibited characteristic secretion profiles, known as the polarization of macrophages [41]. The CM and CCL2 treatment up-regulated the expression of markers associated with classically activated (M1-polarized) macrophages, such as TNF-a, while also increasing the expression of markers for alternatively activated (M2-polarized) macrophages, including CD301 and CD163 (Fig. 4C, Figure S3B). Flowcytometry analysis further revealed an increase in the mean fluorescence intensity (MFI) of CD86 and CD206 following CM and CCL2 induction. This increase was suppressed by treatment with INCB3284, demonstrating CCL2's role in regulating cell activation markers (Fig. 4D, Figure S3C, D). Immunostaining consistently validated the infiltration of M1- and M2-polarized macrophages in lung sections from COPD patients, as evidenced by the detection of Nos2^+^ and Mrc1^+^ macrophages (Fig. 4E, F). Therefore, the role of CCL2 in inducing macrophage activation has been confirmed. The changes in the functions of activated macrophages require further investigation. Matrix metalloproteinase 9 (MMP9) and transforming growth factor beta (TGF-β) were considered important hazardous cytokines in the pathogenesis of COPD, as they respectively participated in the processes of alveolar structure destruction and airway remodeling [42–44]. The RT-qPCR analysis found the elevated mRNA levels of *TGFB1* and *MMP9* in CM-treated THP-1 cells (Fig. 4G). Similarly, this trend was observed in western blot and ELISA assays (Fig. 4H, I). Intriguingly, CM induced alterations in both latent and active forms of TGF-β, whereas CCL2 specifically influenced the levels of active TGF-β (Figure S3F). Furthermore, we observed that CCL2 treatment increased the mRNA levels of *MMP9* and the secretion levels of TGF-β, yet it did not affect the mRNA levels of *TGFB1* (Figure S3E, G). This differential impact might be attributable to other cytokines present in the CM that contribute to TGF-β synthesis. Collectively, these results supported that CCL2 was involved in the recruitment and activation of macrophages, as well as increasing the secretion of COPD pathogenic cytokines.

To further substantiate these conclusions, we extracted BMDM cells from mice and induced them with recombinant mouse-Ccl2 protein. We similarly found that Ccl2 induced upregulation of CD163, CD301, TNF-α, and the active form of TGF-β protein levels in BMDM cells (Figure S3H). RT-qPCR experiments demonstrated that 50 ng/mL Ccl2 treatment significantly increased the expression levels of *Nos2*, *Tnf*, *Cd163*, and *Mrc1* (Figure S3I). Although the change in *Cd86* was not statistically significant, an upward trend was observed (Figure S3I). This significant induction of BMDMs by Ccl2 confirmed the stability of the aforementioned experimental conclusions. Consistent with previous results, Ccl2 did not affect *Tgfb1* mRNA levels (Figure S3E, I).

Additionally, we evaluated other macrophage functions by detecting markers, including RNS generation, immunomodulatory ability, phagocytic activity, and antigen presentation capacity. Among these, only the expression of *NOS2* showed significant changes, indicating that activated macrophages exacerbated local oxidative stress (Figure S3J). However, relying solely on marker detection for functional assessment is somewhat limited; subsequent assays to test specific functions are necessary.

### The activation of macrophages by CCL2 depends on the PI3K-AKT signaling pathway

**Fig. 5:**
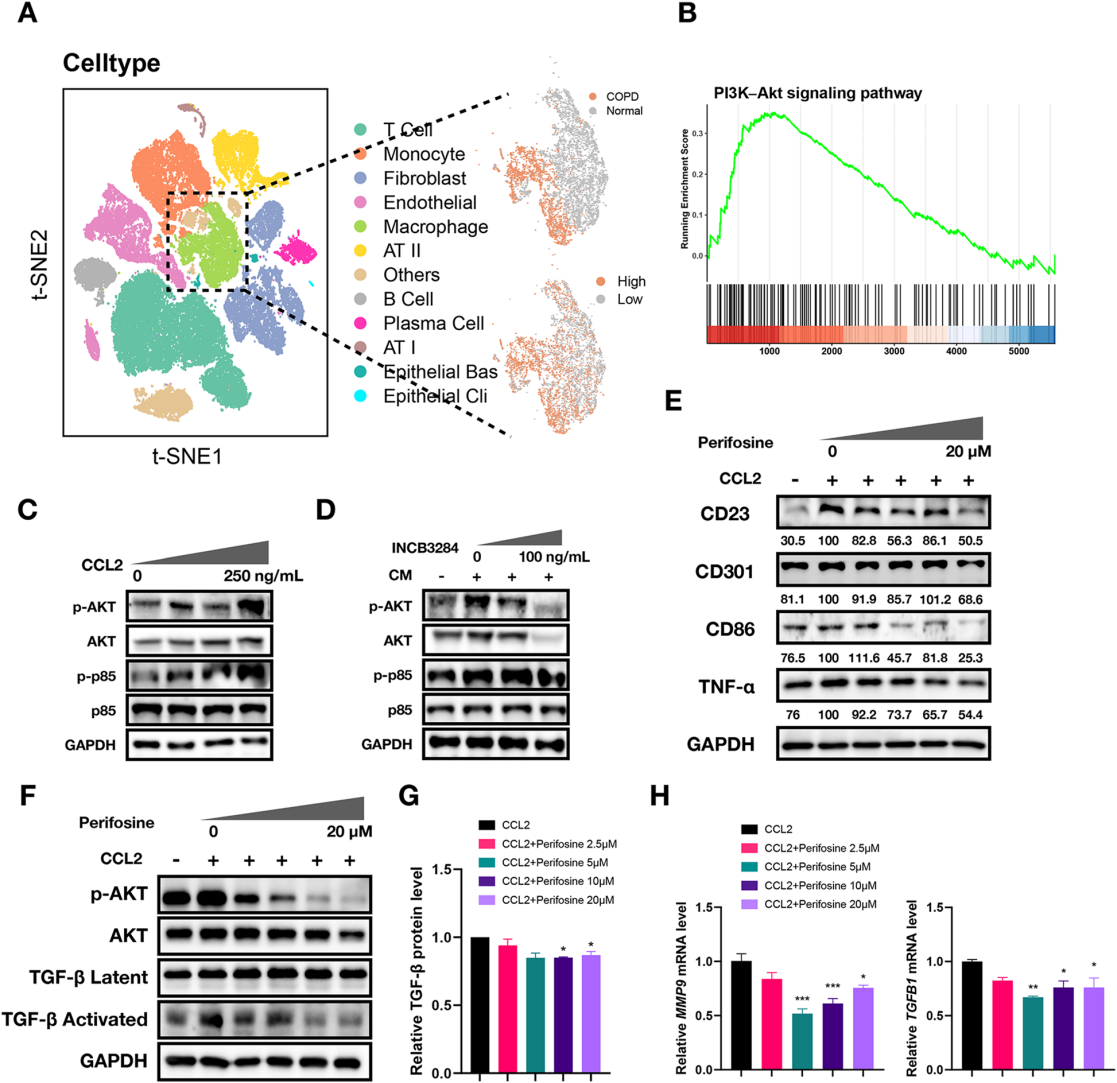
CCR2-mediated macrophage activation depends on the PI3K-AKT signaling pathway. **A** Dimplot illustrated clusters from single-cell data of the GSE196638 dataset, with a focus on macrophage subpopulations. Upon extraction of these subpopulations, a clear segregation between cells originating from COPD and Normal sources was observed. Further analysis using the AUCell method, which calculated the activity of the PI3K-AKT signaling pathway (hsa04151), revealed that cells with high PI3K-AKT signaling (using the median value as a threshold) closely match the distribution of cells from COPD. **B** GSEA plot illustrating the enrichment of DEGs in the PI3K-AKT signaling pathway (hsa04151) in macrophages between COPD and normal groups, indicating a potentially enhanced activation or modulation of this pathway in COPD macrophages. **C**, **D** Western blot assay was conducted to assess the phosphorylation levels of AKT and p85 in THP-1 cells following treatment with CCL2 (**C**) or CM/INCB3284 (**D**). **E** Western blot analysis of activation marker protein levels in THP-1 cells treated with CCL2 and perifosine. Protein band intensities are normalized to GAPDH as a loading control to ensure accurate quantification. The relative expression levels of each activation marker are calculated as ratios compared to the CCL2-treated group, utilizing ImageJ for band intensity analysis. **F** Western blot assay was conducted to assess the phosphorylation levels of AKT and TGF-β in THP-1 cells treated with CCL2 and perifosine. **G** ELISA experiments were conducted to compare the inhibitory effect of perifosine treatment on TGF-β secretion levels induced by CCL2. The data were normalized to the CCL2 group to calculate relative levels. **H** Column plot showing the *MMP9* and *TGFB1* mRNA levels across five cell groups. GAPDH levels were served as loading control

The CCL2-CCR2 axis triggers multiple signaling pathways; however, the pathway that contributes to the pathology of COPD require further analysis [45–47]. Analysis of scRNA data from lung tissues of COPD patients and healthy individuals revealed that macrophages from COPD samples exhibited stronger PI3K-AKT activity (Fig. 5A), and the DEGs were enriched in PI3K-AKT pathways using GSEA (Fig. 5B, Figure S4A, B). We experimentally confirmed the alteration of PI3K/AKT signaling in THP-1 cells treated with CCL2. The levels of phosphorylated p85, which is a PI3K subunit, and phosphorylated AKT (p-p85 and p-AKT) increased in a dose-dependent manner following stimulation with CCL2 (Fig. 5C). In a similar vein, CM treatment elevated the levels of p-p85 and p-AKT, which were inhibited by INCB3284 (Fig. 5D), demonstrating the association of CCL2 with the induction of the PI3K-AKT pathway. We speculate that the induction of macrophage activation by CCL2 depends on PI3K-AKT activity. Perifosine, an AKT inhibitor, reduced the levels of both M1 (CD86, TNFA) and M2 (CD23, CD301) polarization markers in THP-1 cells, suggesting it attenuated macrophage activation (Fig. 5E). This effect was paired with lower *IL6* and *CD163* mRNA levels, further indicating a suppressive effect on macrophage polarization (Figure S4C). The inhibition of AKT phosphorylation by perifosine was notified, which also led to reduced levels of activated form of TGF-β (Fig. 5F). Additionally, both mRNA levels of *MMP9* and *TGFB1* and protein levels of TGF-β were found to be lower in perifosine-treated cells (Fig. 5G, H). Collectively, these results underscore the critical involvement of PI3K-AKT signaling in CCL2-driven macrophage activation.

### Administration of INCB3284 protects mice against CS- and LPS-induced alveolar injury and airway remodeling

**Fig. 6:**
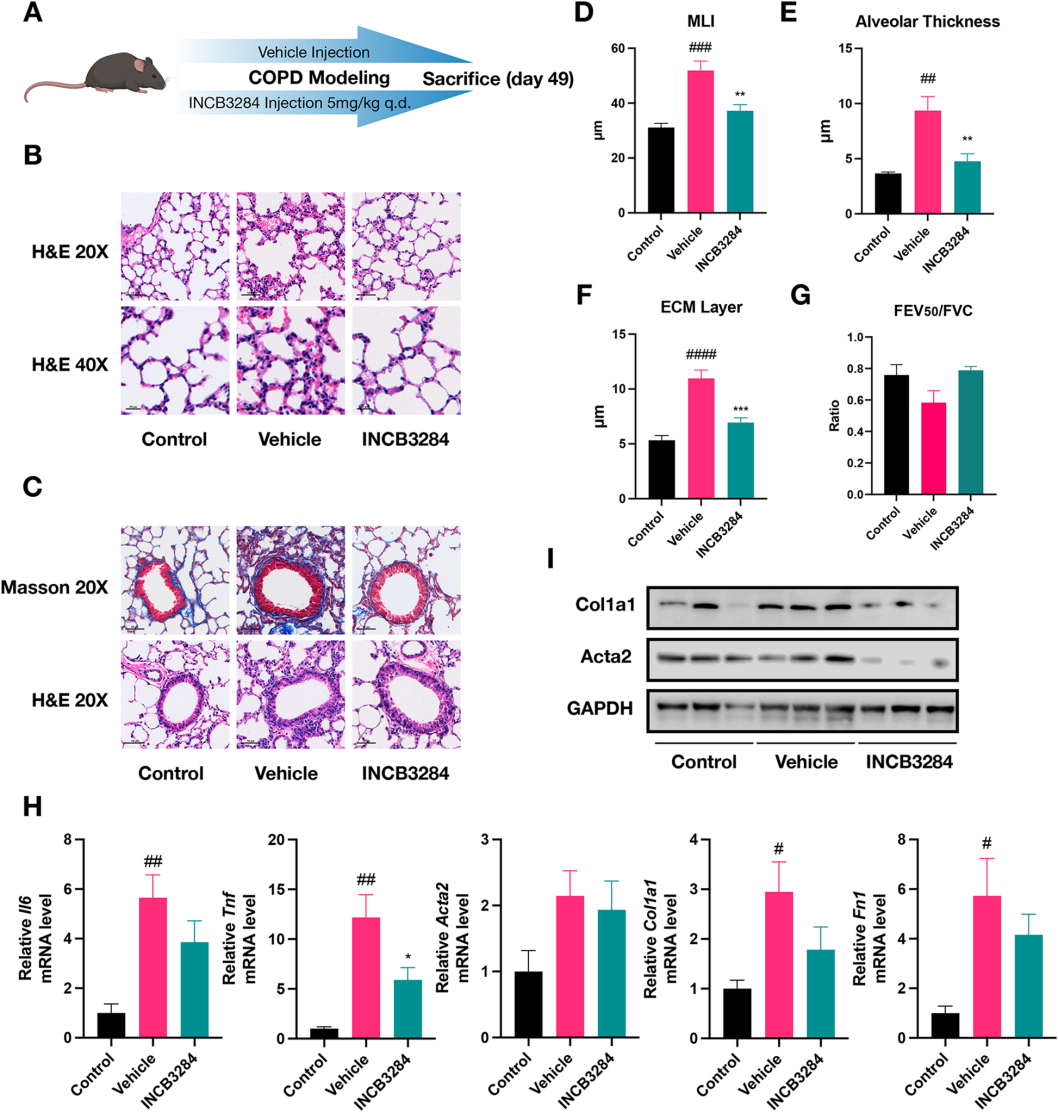
INCB3284 protects mice against CS- and LPS-induced alveolar injury and airway remodeling. **A** Schematic diagram of the INCB3284 treatment experiment. **B** Photos of H&E-stained lung tissue sections from Control, Vehicle and INCB3284-treatment groups. The images were presented at two magnifications: 20X, where the scale bar represents 50 μm, and 40X, where the scale bar represented 20 μm. **C** Photos of H&E- and Masson-stained lung tissue sections from Control, Vehicle and INCB3284-treatment groups. Scale bar: 50 μm. **D**, **E** Column plot displaying the MLI (**D**) and alveolar thickness (**E**) levels across three groups. The symbol '#' denoted statistical comparisons between Control and Vehicle groups, while '*' marks comparisons of INCB3284 and Vehicle groups. The number of symbols correlated with the significance of the *p* values, consistent with previous descriptions. **F** Column chart displaying the thickness of ECM across three groups. **G** Column chart depicting the values of FEV_50_/FVC across three groups. **H** Column graph presents the relative mRNA expression levels of the Il6, Tnf, Acta2, Col1a1, and Fn1 across different groups of mice. Error bars indicated the SEM within each group. **I** Western blot assay was conducted to assess the levels of Col1a1 and Acta2 in lungs derived from the three groups

Finally, our goal is to leverage the insights gained from our findings to formulate a therapeutic strategy for COPD. Targeting and inhibiting disease-associated signaling pathways with compounds is a common strategy in developing therapies. We tried to mitigate pathological injury by targeting the Ccl2-Ccr2 signaling axis in a mouse model utilizing INCB3284. Specifically, mice received daily INCB3284 injections at 5 mg/kg for 49 days (Fig. 6A). INCB3284 treatment notably reduced CS- and LPS-induced alveolar damage and remodeling, as evidenced by H&E and Masson’s staining (Fig. 6B, C). Histopathological alterations were quantitatively assessed using MLI scoring, alveolar thickness, and average ECM layer thickness, revealing a significant alleviation in COPD severity in INCB3284-treated mice (Fig. 6D-F). Additionally, INCB3284-treated mice exhibited improved pulmonary function, shown by an elevated FEV_50_/FVC ratio compared to the vehicle group (Fig. 6G). RT-qPCR analysis further confirmed a substantial decrease in the expression of *Il6*, *Tnf*, *Fn1*, *Col1a1*, and *Acta2* (Fig. 6H), complemented by the downregulation of Col1a1 and Acta2 protein levels in INCB3284-treated lung tissues (Fig. 6I). Thus, INCB3284 significantly countered lung tissue damage, fibrosis, and aberrant respiratory function.

In summary, our findings suggested that INCB3284 administration represents a promising therapeutic approach for COPD, underscoring the CCL2-CCR2 axis as a strategic target for managing the disease.

## Discussion

In the present report, we detailed both in vivo and in vitro studies to explore the CCL2-CCR2 axis's role in COPD pathogenesis. Increased Ccl2 expression was noted in the lungs from COPD mice, where Ccl2 deficiency offered protection against CS- and LPS-induced damage and remodeling. Specifically, CS components and LPS triggered CCL2 production in bronchial epithelial cells by promoting STAT1 phosphorylation, initiating inflammatory responses. Secreted CCL2 engaged CCR2 on macrophages, enhancing their recruitment and activation via the PI3K-AKT pathway. These activated macrophages then secreted high levels of MMP9 and TGF-β, leading to alveolar destruction and remodeling. Our data strongly suggest that inhibiting the CCL2-CCR2 axis can protect against lung injury and remodeling, emphasizing its therapeutic potential for COPD.

The diagnosis of COPD is based on the decline in pulmonary function due to alveolar damage or remodeling. Despite varied pathological features, chronic inflammation and macrophage infiltration consistently appear in COPD lungs. This has sparked researchers' interest in chemokines. Previous studies have highlighted the roles of various chemokines in COPD pathogenesis. CXCL8 is elevated in COPD patients and recruits neutrophils, exacerbating inflammation [48]. CXCL9, CXCL10, and CXCL11, binding to CXCR3, attract Th1 and CD8 + T cells, contributing to immune responses [49]. CXCL12 is involved in stem cell homing and tissue repair but is reduced in COPD, impairing these processes [50]. These studies underscored chemokines' potential roles in COPD, while our work focused on the critical role of CCL2. CCL2's classical function in macrophage recruitment aligns with prior studies [10, 51]. Chronic inflammation induced by cigarette smoke is a persistent cause of COPD progression. Previous research suggested that activated macrophages recruit monocytes through CCL2 secretion, forming a critical positive feedback loop [52]. However, the initial phase of chronic inflammation induction and the role of CCL2 in this process remain unresolved. While CCL2 broadly regulates macrophage function [53], no current studies explicitly describe the relationship between cigarette smoke components, CCL2, and macrophage function in COPD pathogenesis. This study aims to address this gap. Immunofluorescence staining indicated activated macrophage infiltration in COPD mouse lungs, encompassing M1 (Nos2^+^) and M2 (Mrc1^+^) types. CCL2's role in macrophage activation was further supported by in vitro findings. The secretion profiles of activated macrophages, notably MMP9 and TGF-β, underscore their contribution to COPD pathology. MMP9, secreted by macrophages, has been identified as an independent risk factor for COPD, and it plays a pivotal role in the process of alveolar injury [43, 54]. TGF-β was commonly recognized as a classic pro-fibrotic cytokine, and fibrosis was a universal process in airway remodeling. The expression levels of both MMP9 and TGF-β were reported to be significantly elevated in M2-type cells compared to M1-type cells. Additionally, the PI3K-AKT signaling pathway has been identified as a critical molecular regulator of macrophage M2 programming [55]. Similarly, our studies in this report demonstrated that CCL2 induced macrophages activation through PI3K-AKT pathway. In summary, we believed that the M2-like cells might play a more crucial role in inducing COPD pathological changes, while M1-like macrophages mainly participate in maintaining the inflammatory microenvironment.

PI3K-AKT pathway has been reported to influence pulmonary inflammation and oxidative stress [56]. In a PM2.5-induced COPD model, inhibition of this pathway reduced autophagy, promoted epithelial cell apoptosis, and exacerbated alveolar destruction, reflecting the complex effects of this pathway in chronic inflammatory lung diseases [56, 57]. Furthermore, its regulation of macrophage phagocytic function may have potential pathogenic effects. We also detected changes in the expression levels of other important molecular markers in macrophages treated with CCL2. The expression of *NOS2* was significantly elevated, whereas the expression of *TREM2* and *IL10* (immune regulation), *MERTK* (efferocytosis), *CD80*, and *HLA-DRB1* (antigen presentation) did not show significant changes (Figure S3J). Another pathological feature of COPD patients is the impairment of host defense mechanisms, which may be due to epithelial barrier damage and extensive suppression of the innate immune system caused by smoking. This is remarkably similar to the immunophenotype associated with aging [58]. Consequently, the lungs of COPD patients are frequently colonized by pathogens and commonly develop bacterial and viral infections, leading to secondary inflammation. The impact of CCL2 on the phagocytic ability of macrophages warrants further investigation. Although we assessed various functions of macrophages by analyzing mRNA expression levels, transcriptional analysis alone is limited, and more direct experimental detection is necessary.

Exploring CCL2's regulatory mechanisms, we turned to JASPAR and TRRUST databases, identifying STAT1 and STAT3 as potential regulators. Experimental data confirmed CCL2 upregulation via STAT1 phosphorylation in epithelial cells. Hüntelmann et al.'s study demonstrated the direct interaction between STAT1 and the CCL2 promoter region through electrophoretic mobility shift assays (EMSA) and Pull-down Assay [59]. Additionally, our analysis of STAT1 ChIP-Seq data in HeLa cells revealed significant STAT1 binding peaks in the CCL2 promoter region following IFN-γ treatment (Figure S2F). These findings collectively demonstrate the direct regulatory role of STAT1 in CCL2 transcription. Consistent with our findings, Holloway et al. found enhanced phosphorylation of STAT1 in sputum cells of COPD patients [60]. Interestingly, a study by Southworth et al*.* highlighted alveolar macrophages' corticosteroid resistance through the IFNγ-STAT1 pathway, suggesting STAT1's multifaceted role in COPD progression [61].

Based on these data, we applied the inhibitor of CCR2, namely INCB3284, to protect mice against COPD. Although intratracheal administration of INCB3284 offers enhanced precision in treatment, the repeated anesthesia may potentially result in increased mortality and compromised experimental reliability. Therefore, the mice received daily intraperitoneal injection of INCB3284. As a result, delivery of INCB3284 protected mice from CS and LPS-induced lung injury and remodeling. The typical pharmacological strategy for COPD includes bronchodilators and anti-inflammatory drugs. Recent drug development for COPD focuses on targets related to specific inflammatory proteins, such as CXCR2 and MAPK, but their therapeutic effects are limited [62]. Currently, over 30 CCR2 inhibitors are in development for various malignancies and inflammatory diseases. These inhibitors show promise based on CCR2's role in cell migration to inflammation and tumor sites. Preclinical studies indicated that CCR2 inhibitors significantly reduced tumor metastasis and mortality in animal models [63, 64]. Clinical trial of CCX872-B for pancreatic cancer demonstrated good oral tolerance and weak efficacy in tumor control [65]. A preclinical meta-analysis suggested that targeting CCL2 or CCR2 with pharmacological treatments may reduce the burden of atherosclerotic lesions [66]. Additionally, in autoimmune diseases, CCR2 inhibitors effectively reduce inflammation, demonstrating their potential as therapeutic agents. In clinical trial, AZD-2423, tested for COPD, showed no significant adverse events, with mild side effects like dry mouth and headache. However, the compound did not demonstrate excellent therapeutic effects (NCT01153321). Researchers believe that the insufficient efficacy of various CCR2 drugs is related to their inability to effectively block the CCL2-CCR2 axis for a sufficiently long duration [67]. Moreover, given the high heterogeneity of COPD, inhibiting a single target may not be sufficient to address the characteristics of multiple patient subgroups. Therefore, combination therapy is a potential approach. Considering patients' good tolerance to CCR2 inhibitors, this target has the potential for developing long-term combination therapy strategies. To address these issues, we will first discuss the therapeutic effects, toxicology, and pharmacokinetic characteristics of combination therapies involving CCR2 inhibitors and traditional drugs in vivo based on existing conclusions and attempt to apply for clinical trial approval. On the other hand, developing more potent CCR2 inhibitors is also a future direction. The development of CCR2 inhibitors often faces numerous challenges, including selectivity for other chemokine receptors and ion channels. Future research will focus on developing combination therapy strategies and synthesizing more effective compounds for CCR2 inhibitors in COPD treatment.

This study has the following limitations: Although the study discussed gene expression changes in macrophages stimulated by CCL2, a comprehensive investigation into their functional alterations is lacking. Besides, this study proposed that the CCR2-PI3K-AKT signaling pathway served as a crucial pathway for CCL2-induced activation. However, it is imperative to validate the phenotypic alterations of macrophages upon inhibition of PI3K and AKT activation through in vivo research. Future research will address these limitations by conducting detailed experiments to quantify the phagocytic capacity of macrophages, antigen processing efficiency, and examining the ability of macrophages to clear apoptotic cells (efferocytosis). Additionally, we will employ genetic and pharmacological approaches to inhibit PI3K and AKT activation in vivo, followed by phenotypic analyses to observe the resultant effects on macrophage behavior and function. These studies will include evaluating the impact of pathway inhibition on macrophage recruitment, polarization, and their role in disease models. Through these research plans, we aim to provide a more comprehensive understanding of the functional alterations in macrophages induced by CCL2 and the pivotal role of the CCR2-PI3K-AKT signaling pathway in these processes.

In conclusion, this study sheds light on the regulatory role of STAT1 in the induction of CCL2 secretion in epithelial cells subjected to CS and LPS. We have also elucidated that the activation of the CCL2-CCR2-PI3K-AKT signaling cascade plays a pivotal role in macrophage activation, which significantly contributes to lung injury and airway remodeling. Notably, our research reveals that the inhibition of CCR2 with INCB3284 markedly safeguards mice against alveolar damage and airway remodeling. These findings underscore the significant therapeutic promise of targeting the CCL2-CCR2 axis as a novel strategy in the treatment of COPD.

### Supplementary Information

Supplementary Material 1.

Supplementary Material 2.

## Data Availability

The datasets used and/or analysed during the current study are available from the corresponding author on reasonable request. The graphic abstract plot was generated with BioRender with the license for publication usage (LF2706T9L5).

## Abbreviation

COPD: Chronic obstructive pulmonary disease
CCL2: C-C motif chemokine ligand 2
CCR2: C-C motif chemokine receptor 2
PI3K: Phosphoinositide 3-kinase
STAT1: Signal transducer and activator of transcription 1
KO: Knockout
LPS: Lipopolysaccharide
FBS: Fetal bovine serum
PMA: Phorbol 12-myristate 13-acetate
CS: Cigarette smoke
CSE: Cigarette smoke extract
ELISA: Enzyme-linked immunosorbent assay
MMP9: Matrix metalloproteinase 9
TGF-β: Transforming growth factor beta
ECM: Extracellular matrix
MLI: Mean linear intercept
DEGs: Differentially expressed genes
GSEA: Gene set enrichment analysis
GO: Gene ontology
TFs: Transcription factors
IF: Immunofluorescence
RT-Qpcr: Reverse transcription quantitative polymerase chain reaction
scRNA-Seq: Single-cell RNA sequencing
FEV50/FVC: Forced expiratory volume in 50 ms/forced vital capacity
IL1B: Interleukin 1 beta
IL6: Interleukin 6
