# Supplementary material for: Targeting CCL2-CCR2 signaling pathway alleviates macrophage dysfunction in COPD via PI3K-AKT axis
Source: Cell Commun Signal. 2024 Jul 17;22:364. doi: 10.1186/s12964-024-01746-z (PMC11253350; doi:10.1186/s12964-024-01746-z)
Supplement: Supplementary file 1 — Supplementary Material 1. [file 12964_2024_1746_MOESM1_ESM.docx]

**Targeting CCL2-CCR2 Signaling Pathway Alleviates Macrophage Dysfunction in COPD via PI3K-AKT Axis**

**Supplemental Tables**

***Table S1. List of Used Antibosies***

| Antibody | Company | Cat. |
| --- | --- | --- |
| CCL2 | Abclonal | A7277 |
| EpCAM | Abclonal | A19301 |
| PECAM1 | Abclonal | A0378 |
| Fibronectin | Abclonal | A16678 |
| p-STAT1 | CST | 9167 |
| STAT1 | Abclonal | A19563 |
| GAPDH | CST | 2118 |
| CD163 | Abmart | TD8235S |
| CD301 | Abclonal | A11859 |
| CD86 | Abclonal | A1199 |
| TNF-α | Abclonal | A22227 |
| CD68 | Abclonal | A15037 |
| NOS2 | Abmart | T55993S |
| TGF-β | Abmart | PA2154S |
| p-AKT | CST | 4060S |
| AKT | CST | 4691S |
| p-p85 | Abmart | T40116S |
| p85 | Abclonal | A4992 |
| CD23 | Abclonal | A23092 |
| COL1A1 | CST | 72026 |
| ACTA2 | CST | 19245 |
| MRC1 | Abmart | TD4149S |
| CD86-APC | Biolegend | 305411 |
| CD206-PE | BD | 555954 |

***Table S2. Sequences of Primers***

| Species | Gene | Primer seq |
| --- | --- | --- |
| mouse | *Ccl2-F* | TTAAAAACCTGGATCGGAACCAA |
| mouse | *Ccl2-R* | GCATTAGCTTCAGATTTACGGGT |
| mouse | *Il6-F* | TACCACTTCACAAGTCGGAGGC |
| mouse | *Il6-R* | CTGCAAGTGCATCATCGTTGTTC |
| mouse | *Tnf-F* | GGTGCCTATGTCTCAGCCTCTT |
| mouse | *Tnf-R* | GCCATAGAACTGATGAGAGGGAG |
| mouse | *Acta2 -F* | TGCTGACAGAGGCACCACTGAA |
| mouse | *Acta2 -R* | CAGTTGTACGTCCAGAGGCATAG |
| mouse | *Col1a1-F* | CCTCAGGGTATTGCTGGACAAC |
| mouse | *Col1a1-R* | CAGAAGGACCTTGTTTGCCAGG |
| mouse | *Fn1-F* | CCCTATCTCTGATACCGTTGTCC |
| mouse | *Fn1-R* | TGCCGCAACTACTGTGATTCGG |
| mouse | *Gapdh-F* | CATCACTGCCACCCAGAAGACTG |
| mouse | *Gapdh-R* | ATGCCAGTGAGCTTCCCGTTCAG |
| human | *CCL2-F* | AGAATCACCAGCAGCAAGTGTCC |
| human | *CCL2-R* | TCCTGAACCCACTTCTGCTTGG |
| human | *MMP9-F* | GCCACTACTGTGCCTTTGAGTC |
| human | *MMP9-R* | CCCTCAGAGAATCGCCAGTACT |
| human | *TGFB1-F* | TACCTGAACCCGTGTTGCTCTC |
| human | *TGFB1-R* | GTTGCTGAGGTATCGCCAGGAA |
| human | *IL6-F* | CCTGAACCTTCCAAAGATGGC |
| human | *IL6-R* | TTCACCAGGCAAGTCTCCTCA |
| human | *CD163-F* | GAGGAGACCTGGATCACATGTGA |
| human | *CD163-R* | GACCACAGCCAAGTTGTTGACACAC |
| human | *STAT1-F* | ATGGCAGTCTGGCGGCTGAATT |
| human | *STAT1-R* | CCAAACCAGGCTGGCACAATTG |
| human | *STAT3-F* | CAGCAGCTTGACACACGGTA |
| human | *STAT3-R* | AAACACCAAAGTGGCATGTGA |
| human | *GAPDH-F* | GTCTCCTCTGACTTCAACAGCG |
| human | *GAPDH-R* | ACCACCCTGTTGCTGTAGCCAA |

***Table S3. TF Prediction with JASPAR and TRRUST***

| JASPAR TF List | TRRUST TF List |
| --- | --- |
| ZNF816 | APEX1 |
| SOX21 | ATF4 |
| NFIB | CEBPA |
| STAT4 | HDAC2 |
| ZNF460 | IRF3 |
| NFATC4 | JUN |
| NFATC3 | NFAT5 |
| ELF3 | NFIC |
| GABPA | NFKB1 |
| EHF | NFKB1 |
| STAT1 | NR1I2 |
| STAT3 | PREB |
| FOXH1 | REL |
| FOXO1 | RELA |
| FOXI1 | RELA |
| ELF1 | SP1 |
|  | SP1 |
|  | SPI1 |
|  | STAT1 |
|  | STAT2 |
|  | STAT3 |
|  | XBP1 |
