# Supplementary material for: Targeting CCL2-CCR2 signaling pathway alleviates macrophage dysfunction in COPD via PI3K-AKT axis
Source: Cell Commun Signal. 2024 Jul 17;22:364. doi: 10.1186/s12964-024-01746-z (PMC11253350; doi:10.1186/s12964-024-01746-z)
Supplement: Supplementary file 2 — Supplementary Material 2. [file 12964_2024_1746_MOESM2_ESM.docx]

**Targeting CCL2-CCR2 Signaling Pathway Alleviates Macrophage Dysfunction in COPD via PI3K-AKT Axis**

**Supplemental Figures**

**
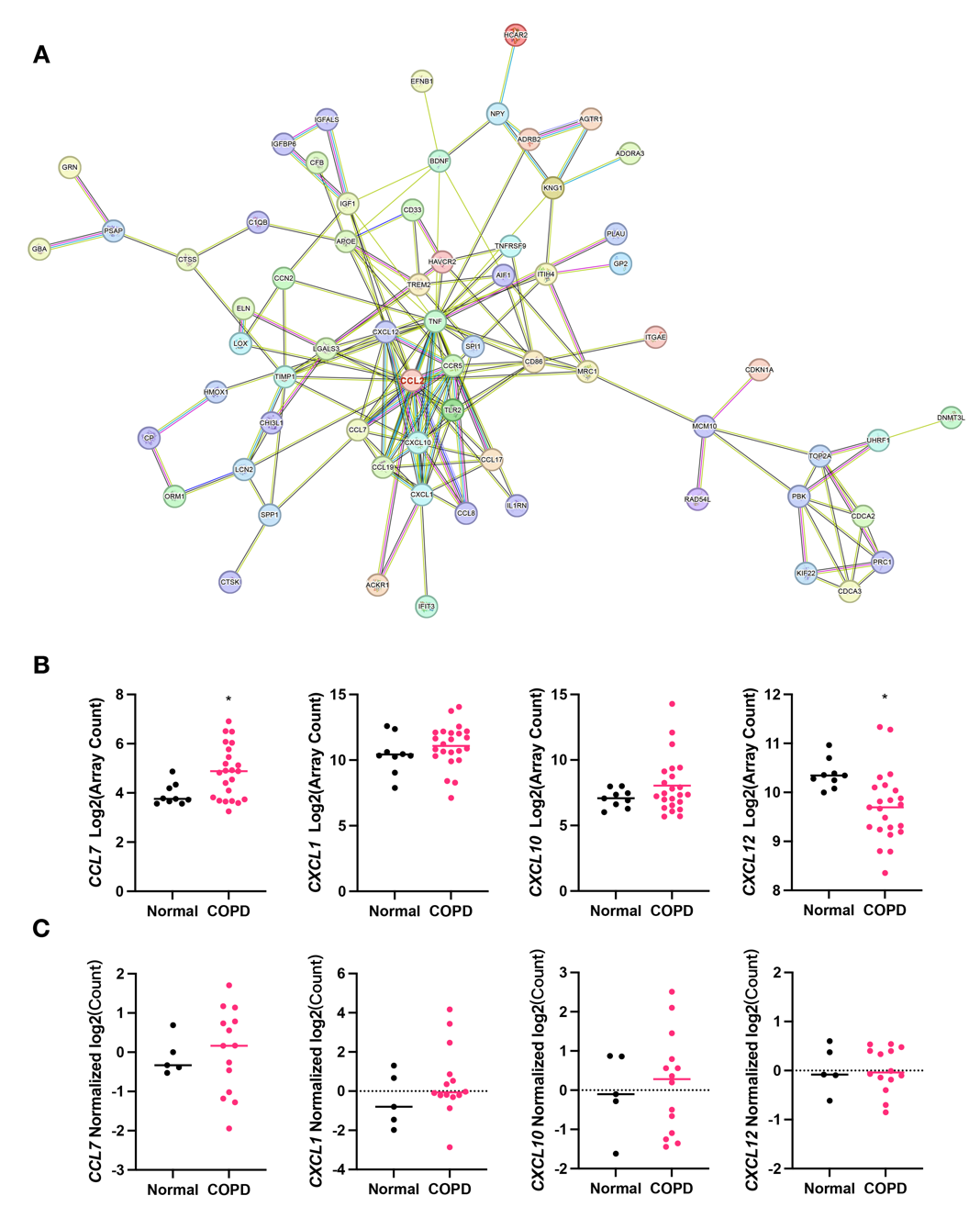
**

**Figure S1. Analysis of chemokine profiles**

(A) Network diagram of protein-protein interaction analysis for DEGs (GSE52509), highlighting CCL2 at a central position.

(B, C) Scatter plots of CCL7, CXCL10, CXCL1 and CXCL12 mRNA levels, respectively, based on datasets GSE38974 (B) and GSE106986 (C). Horizontal lines represented the mean values. Significance was determined through t-tests, with notations indicating levels of significance: * for p < 0.05, ** for p < 0.01, *** for p < 0.001, and **** for p < 0.0001. The absence of a marker denoted a lack of significance.

**
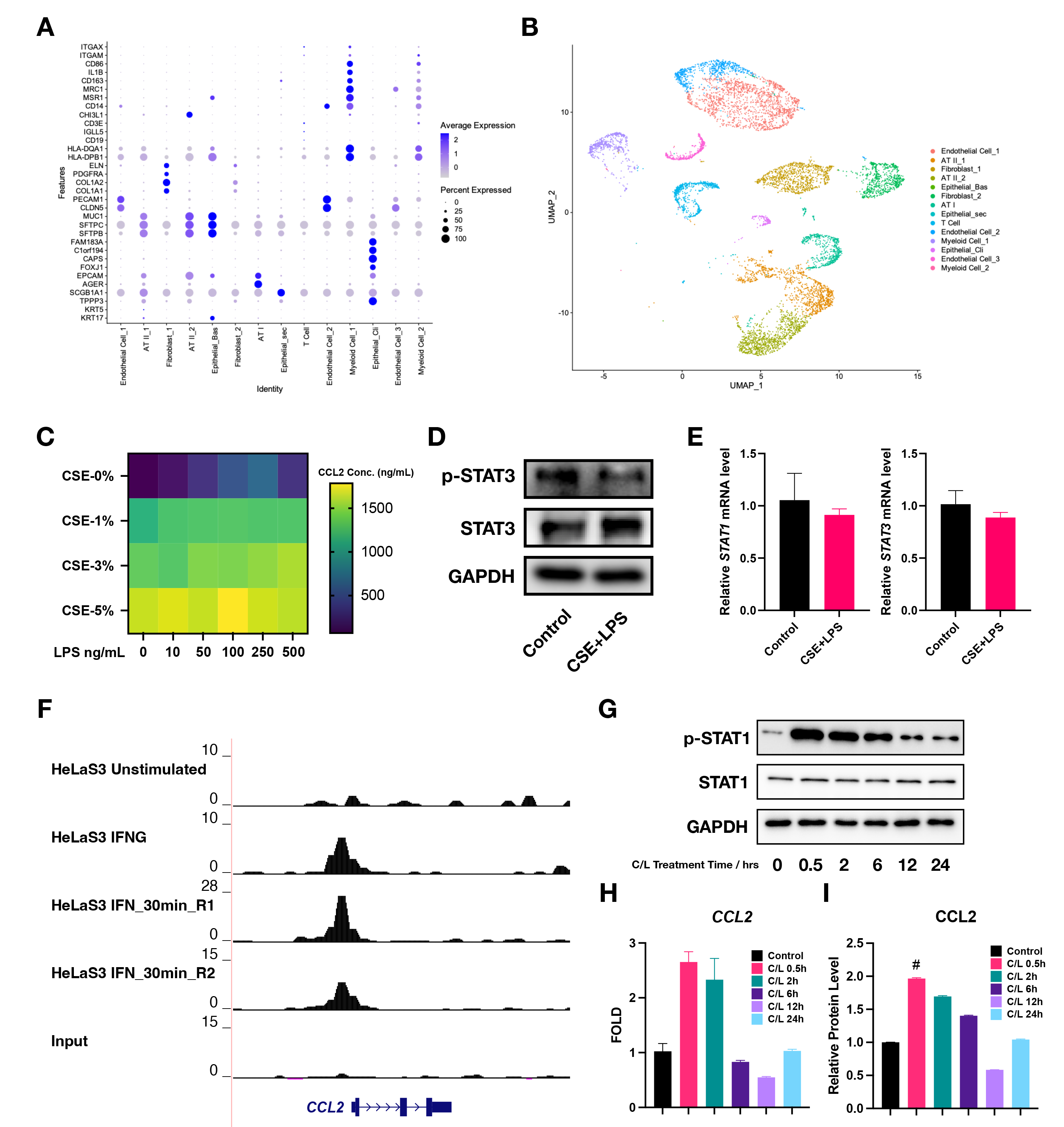
**

**Figure S2. Upstream mechanisms of CCL2 expression regulation**

(A) Dotplot was depicted for annotating clusters from single-cell data, where deeper colors indicated higher average expression levels, and larger dots represented a higher proportion of cells within the cluster expressing the gene.

(B) Dimplot of UMAP-reduced data with cluster annotations.

(C) Heatmap showing CCL2 protein levels in the supernatant of BEAS2B cells after combined treatment with various concentrations of CSE and LPS.

(D) Western blot experiment analyzed changes in STAT3 phosphorylation levels after treatment with CSE+LPS.

(E) Column chart indicated that there are no significant changes in mRNA levels of *STAT1* and *STAT3* following CSE+LPS treatment.

(F) Signal plots showing *CCL2* gene region STAT1-ChIP signals in HeLaS3 cells under various conditions, indicating direct interaction between STAT1 and *CCL2* promotor sequence.

(G) Western blot experiment showing changes in STAT1 phosphorylation levels at various time points after C/L treatment.

(H) Fold changes in *CCL2* expression levels in response to C/L treatment at different time points.

(I) Relative protein levels of CCL2 at different time points following C/L treatment.


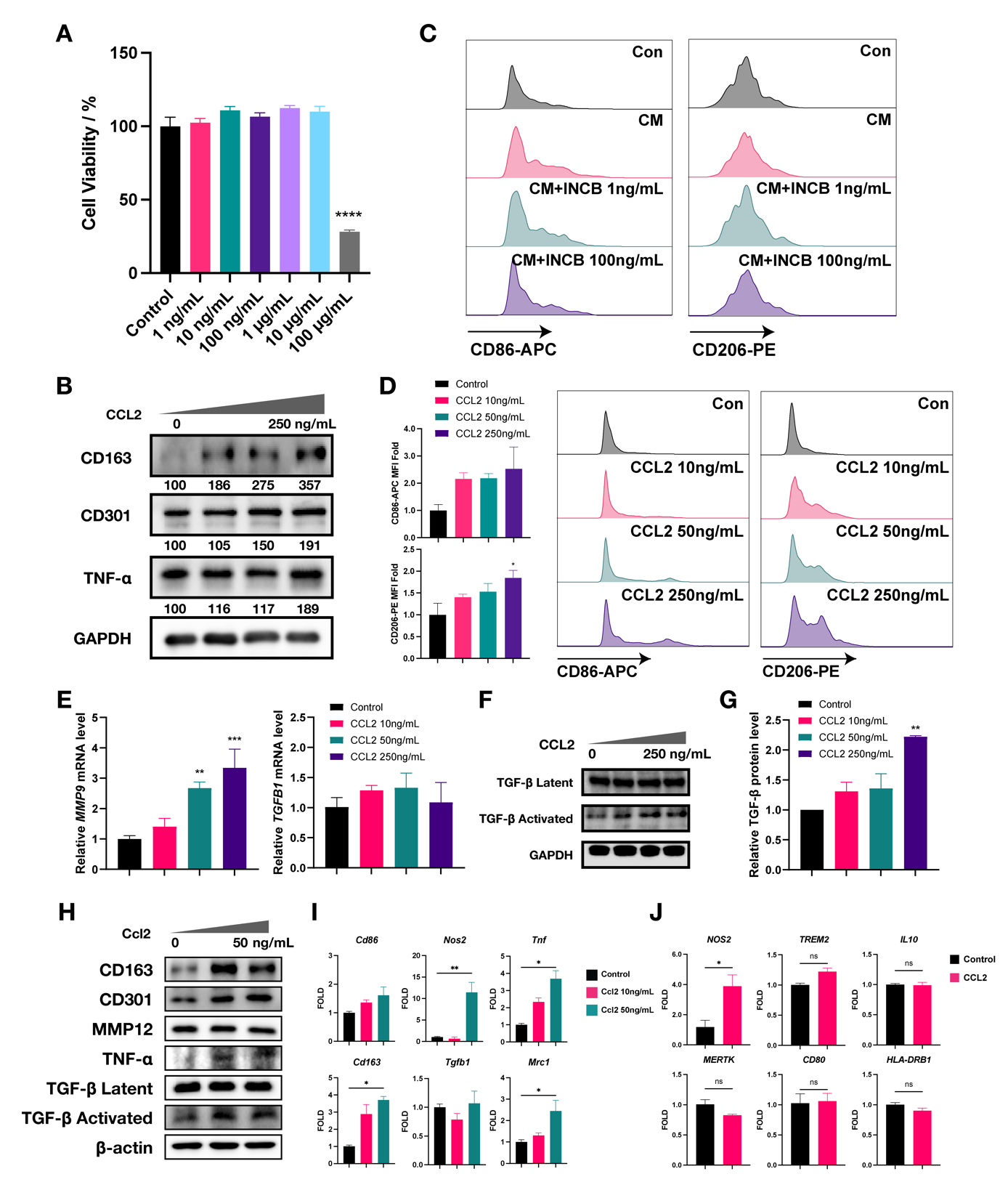


**Figure S3. Induction of macrophage activation by CCL2**

(A) Bar graph depicting cell viability percentage in response to increasing concentrations of CCL2. Cell viability decreased significantly at the highest concentration (1000 ng/mL) compared to control (***: p<0.001).

(B) Western blot analysis showing the expression of macrophage activation markers CD163 CD301 and TNF-α after CCL2 treatment. Band intensities were normalized to GAPDH. The relative expression levels of each activation marker were calculated as ratios compared to the control group, utilizing ImageJ for band intensity analysis.

(C) Flow cytometry histograms comparing the surface expression of CD86 and CD206 on cells treated with CM alone or in combination with INCB3284.

(D) Column graphs and flow cytometry histograms representing MFI ratios of CD86 and CD206 in cells treated with varying concentrations of CCL2.

(E) RT-qPCR graphs displaying the relative mRNA levels of *MMP9* and *TGFB1* after treatment with different concentrations of CCL2.

(F) Western blot images showing levels of latent and activated TGF-β after treatment with CCL2.

(G) Column graph indicating the relative levels of TGF-β protein measured by ELISA, showing a dose-dependent increase after treatment with increasing concentrations of CCL2.

(H) Western blot analysis showing expression levels of CD163, CD301, MMP12, TNF-α, latent and activated TGF-β, and β-actin in BMDMs after treatment with different concentrations of Ccl2.

(I) Column graphs showing fold changes in the BMDMs’ expression levels of *Cd86*, *Nos2*, *Tnf*, *Cd163*, *Tgfb1*, and *Mrc1* in response to different concentrations of Ccl2.

(J) Column graphs showing fold changes in the THP-1 cells’ expression levels of *NOS2*, *TREM2*, *IL10*, *MERKT*, *CD80*, and *HLA-DRB1* in response to CCL2.


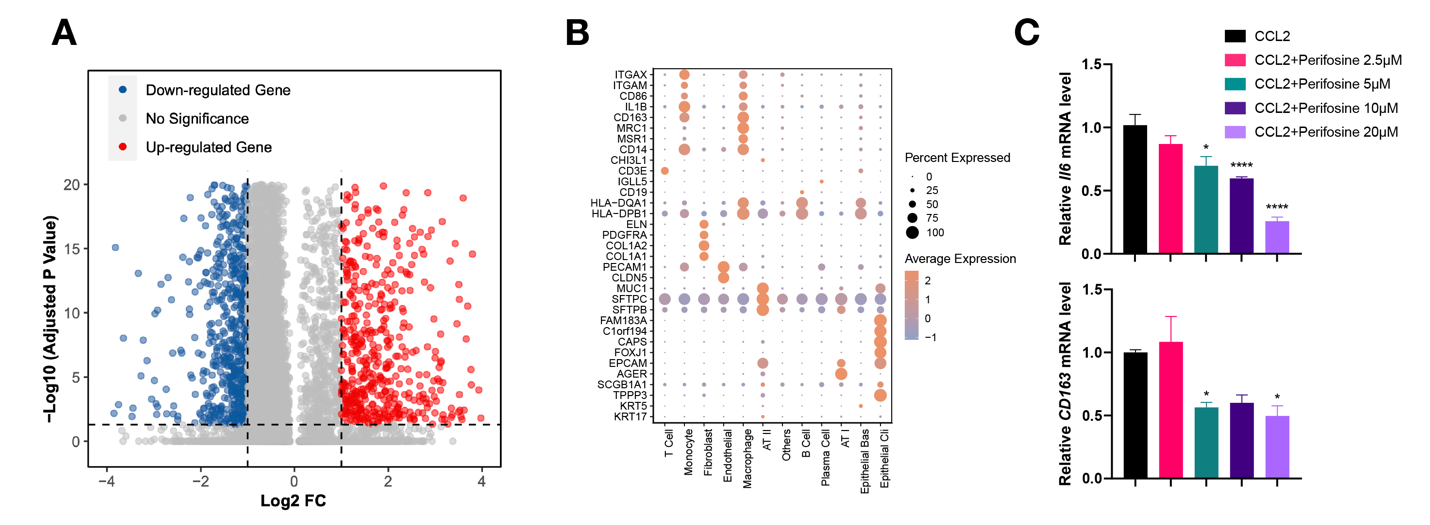


**Figure S4. The activation of macrophages by CCL2 depends on the PI3K-AKT signaling pathway**

(A) Volcano plot highlighting differentially expressed genes between macrophages from COPD and Normal groups. Up-regulated genes are marked in red, down-regulated genes in blue, and genes with no significant change in gray. The x-axis represents log2 fold change (FC), while the y-axis shows the negative logarithm of the adjusted p-value (-log10 adjusted p-value).

(B) Dotplot showing the percentage of cells expressing specific genes (size of dots) and the average expression level (color intensity) across different clusters identified within the scRNA-Seq data.

(C) Bar graphs depicting the relative mRNA levels of *IL6* and *CD163* after treatment with CCL2 alone or in combination with various concentrations of perifosine.
